# Understanding Metabolic Pathway Rewiring by Oncogenic Gamma Herpesvirus

**DOI:** 10.4014/jmb.2407.07039

**Published:** 2024-08-30

**Authors:** Un Yung Choi, Seung Hyun Lee

**Affiliations:** 1 Department of Microbiology, Konkuk University School of Medicine, Chungju 27478, Republic of Korea; 2 KU Open Innovation Center, Research Institute of Medical Science, Konkuk University School of Medicine, Chungju 27478, Republic of Korea

**Keywords:** Metabolic reprogramming, EBV, KSHV, oncogenic virus, metabolic therapeutics

## Abstract

Gamma herpesviruses, including Epstein-Barr virus (EBV) and Kaposi’s sarcoma-associated herpesvirus (KSHV), are key contributors to the development of various cancers through their ability to manipulate host cellular pathways. This review explores the intricate ways these viruses rewire host metabolic pathways to sustain viral persistence and promote tumorigenesis. We look into how EBV and KSHV induce glycolytic reprogramming, alter mitochondrial function, and remodel nucleotide and amino acid metabolism, highlighting the crucial role of lipid metabolism in these oncogenic processes. By understanding these metabolic alterations, which confer proliferative and survival advantages to the virus-infected cells, we can identify potential therapeutic targets and develop innovative treatment strategies for gamma herpesvirus-associated malignancies. Ultimately, this review underscores the critical role of metabolic reprogramming in gamma herpesvirus oncogenesis and its implications for precision medicine in combating virus-driven cancers.

## Introduction

Tumorigenesis, the complex process leading to cancer development, often involves the influence of various pathogens, with viruses playing a prominent role [1]. Among the diverse group of viruses implicated in oncogenesis, gamma herpesviruses are known to be significant contributors to transformation of normal cells into cancerous ones by manipulating the host cellular machinery [2]. Gamma herpesviruses, a subfamily within the Herpesviridae family, comprise well-known human pathogens such as EBV and KSHV. These viruses are characterized by their ability to establish latent infections, persisting within the host for extended periods and occasionally reactivating to cause disease [3]. Both EBV and KSHV maintain their genomes as episomes, which means they exist as circular DNA molecules within the nucleus, allowing them to stay in the infected cells indefinitely and contributing to their oncogenic potential [4].

Gamma herpesviruses not only disrupt fundamental cellular processes but also trigger distinctive metabolic rewiring, a hallmark of cancer [5]. This remodeling influences cellular energy production, biomass synthesis, and redox balance. By identifying specific metabolic vulnerabilities associated with gamma herpesvirus-induced cancers, we can enhance our understanding of the fundamental processes driving cancer progression by oncogenic viruses and pave the way for the development of innovative treatment strategies. This review will provide an overview of the unique metabolic dependencies in gamma herpesvirus-associated cancers, offering new opportunities for precision medicine in the battle against virus-associated cancers.

## Life Cycle and Pathogenesis in Gamma Herpesviruses

Gamma herpesviruses exhibit a biphasic life cycle comprising latent and lytic phases. During the latent phase, the virus persists within the nucleus of host cells as an episome [6]. Latent infection is characterized by the expression of a limited set of viral genes, which ensures maintenance of the viral genome and modulates host cellular mechanisms [7]. For instance, KSHV expresses latency-associated nuclear antigen (LANA), v-cyclin, and viral FLICE-inhibitory protein (vFLIP), which play crucial roles in maintaining latency and contributing to oncogenic processes [8]. In the case of EBV, latency is marked by the expression of latency proteins including the Epstein-Barr nuclear antigens (EBNA) and latent membrane proteins (LMPs), as well as EBV-encoded RNAs (EBERs), such as EBER1 and EBER2 [9]. EBV adopts various latency programs (Latency 0, I, II, and III), each associated with specific types of cells and distinct malignancies. For example, Burkitt’s lymphoma is primarily linked to Latency I, whereas Nasopharyngeal carcinoma (NPC) is associated with Latency II [10].

Reactivation from latency to the lytic phase can be induced by several stimuli, including immune suppression or inflammatory signals. During the lytic cycle, there is a cascade of viral gene expression which results in the production of viral particles, cell lysis, and dissemination of the virus [11]. Additionally, the viral life cycle is influenced by the nutritional and metabolic status of the cell's surroundings. For KSHV, spontaneous reactivation is more frequent in three-dimensional (3D) culture environments, which impose greater metabolic stress [12]. In EBV-associated cancers, changes in the viral life cycle have been observed in response to the supply of metabolites, such as methionine [13]. This reactivation process facilitates the spread of the virus within the host and to new hosts, linking the latent and lytic phases in a cycle that supports both viral persistence and propagation.

EBV infection is highly prevalent, with over 90% of adults worldwide being seropositive. Primary infection typically occurs in childhood and is usually asymptomatic, although in adolescents and young adults, it can manifest as infectious mononucleosis. EBV is implicated in the pathogenesis of several malignancies, including Burkitt’s lymphoma, NPC, and Hodgkin’s lymphoma [14]. The oncogenic potential of EBV is mediated through the expression of its viral genes, which manipulate key cellular pathways involved in apoptosis, cell cycle regulation, and immune evasion, thereby promoting cellular transformation and proliferation [15]. KSHV is similarly implicated in the pathogenesis of several malignancies, most notably Kaposi’s sarcoma (KS), primary effusion lymphoma (PEL), and multicentric Castleman’s disease (MCD). KSHV encodes various oncoproteins and microRNAs that modulate host signaling pathways related to angiogenesis, immune response, and cell survival, thereby contributing to cellular transformation and oncogenesis [16]. The association of KSHV with HIV/AIDS is particularly significant, as immunocompromised individuals, such as those with untreated HIV, are at a considerably higher risk of developing KS. HIV-induced immune suppression facilitates the reactivation and persistence of KSHV and exacerbates the inflammatory environment conducive to cancer development. Moreover, HIV infection alters the metabolic landscape in a way that may support the survival and proliferation of KSHV-infected cells [17, 18]. This interplay between HIV and KSHV creates a synergistic effect that accelerates oncogenesis, leading to more aggressive and widespread disease manifestations in AIDS patients [19].

## Metabolic Alterations in Cancer

Metabolic reprogramming is a hallmark of cancer, characterized by alterations in cellular energy metabolism to support the heightened demands of rapidly proliferating cells. Cancer cells often exhibit elevated glucose uptake, directing glucose towards glycolysis even in aerobic condition, a phenomenon known as the Warburg effect [20]. This provides rapid ATP generation and supports the synthesis of macromolecules required for cell growth [21]. The Warburg effect is further characterized by increased lactate production, contributing to the acidic tumor microenvironment (TME), promoting multiple critical oncogenic processes including tissue invasion/metastasis, angiogenesis, and drug resistance [22, 23]. Numerous cancers exhibit mutations in critical enzyme of tricarboxylic acid (TCA) cycle, including isocitrate dehydrogenase (IDH), succinate dehydrogenase (SDH), and fumarate hydratase (FH) [24]. These mutations contribute to the disruption of TCA cycle integrity, with intermediates to be redirected towards biosynthetic pathways that lead to the production of precursors essential for nucleotide and amino acid synthesis or oncometabolite facilitating tumorigenesis [25]. As a result, cancer cells gain benefits from transforming the TME into a more conducive environment for cancer progression, swiftly synthesizing ATP, and obtaining essential cellular building blocks, albeit at the expense of less efficient ATP production.

As proliferating cells have an increased demand for building block, cancer cells upregulate the Pentose Phosphate Pathway (PPP) and biosynthesis of non-essential amino acids (NEAAs) for nucleotide and protein synthesis, respectively [26]. Additionally, cancer cells frequently exhibit enhanced de novo lipogenesis to synthesize fatty acids for membrane biogenesis and energy storage. Dysregulated lipid metabolism is associated with aggressive phenotypes and metastasis [27]. These metabolic demands and remodeling of these cancer cells are inevitably affected by nutrient competition and the crosstalk with TME. Consequently, under conditions of limited nutrients, cancer cells may employ mechanisms such as autophagy or macropinocytosis to secure their supply of fatty acid or amino acid [28-30]. Surrounding stromal cells further contributes to tumor progression, as evidenced by studies showing that in pancreatic cancer, stellate cells in TME provide alanine through autophagic secretion [31], and in ovarian cancer, adipocytes serve as a source of fatty acids for the cancer cells [32].

In the realm of cancer therapeutics targeting metabolism, CB-839, a small molecule inhibitor specifically designed to target glutaminase (GLS), strategically manipulate the reliance of cancer cells on glutamine, is noteworthy [33, 34]. Similarly, the scrutiny of ivosidenib, a pharmaceutical agent developed for the treatment of relapsed or refractory myelodysplastic syndromes (MDS) characterized by IDH1 mutations [35], provides a compelling motivation for investigating metabolic rewiring mechanisms within diverse cancer types. In the subsequent sections of this review article, we will explore the intricacies of metabolic remodeling induced by gamma herpesviruses. It is anticipated that these insights will contribute to the establishment of a solid foundation for the development of innovative and targeted treatment strategies.

## Oncogenic Mechanisms of Gamma Herpesviruses

### Glycolytic Reprogramming

**Fig. 1:**
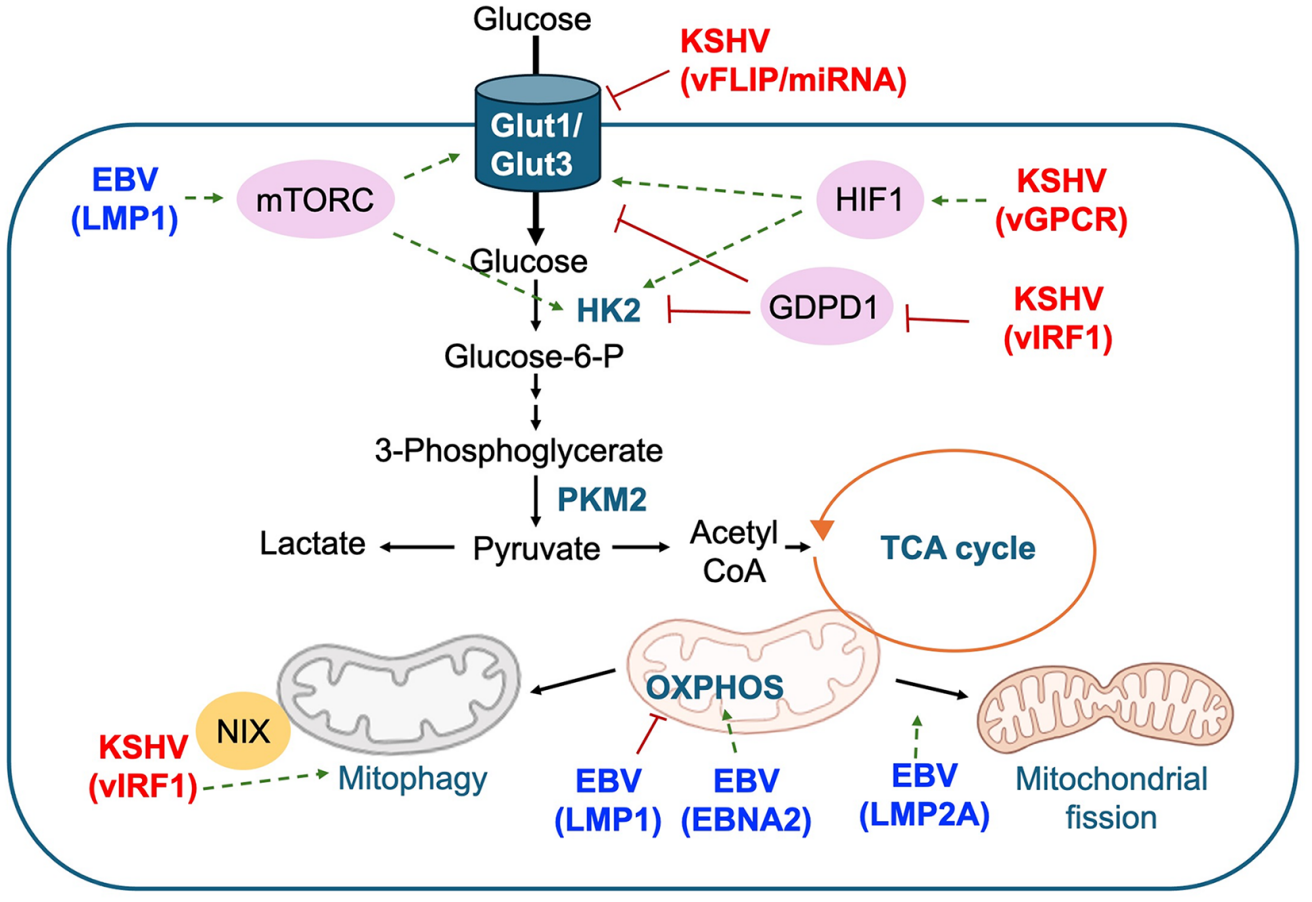
Glycolytic and mitochondrial reprogramming by gamma herpesviruses. EBV and KSHV modulate numerous signaling pathways impacting glycolytic and mitochondrial metabolism. The EBV-encoded latent gene product, LMP1, influences glucose metabolism via several pathways, including mTOR. Similarly, KSHV infection affects glucose metabolism by regulating certain transcription factors, such as HIF1, which activates glycolytic enzymes. Additionally, EBV's LMP1, LMP2A, and EBNA2 are involved in the regulation of OXPHOS and mitochondrial fission. KSHV infection further induces vIRF1-mediated mitophagy. Green dashed lines indicate activation of transcription or activity, whereas red solid lines indicate inhibition of transcription or activity.

EBV induces significant increases in glucose uptake and expression of glycolytic enzymes at both mRNA and protein levels (Fig. 1). LMP1, a transmembrane protein with potent cell signaling properties, induces metabolic reprogramming by activating aerobic glycolysis and triggering essential signaling pathways. Activation of mTORC1 by LMP1 emerges as a key modulator for NF-κB signaling, mediating aerobic glycolysis in NPC cells. This metabolic shift involves upregulation of glucose transporter 1 (Glut-1) transcription, promoting cell growth [36]. Additionally, LMP1-driven metabolic reprogramming contributes to NPC metastasis through the activation of insulin-like growth factor 1 (IGF1)-mTORC2 signaling. This pathway involves the secretion of IGF1, phosphorylation of IGF1 receptor (IGF1R), and activation of mTORC2/AKT signaling, linking glucose metabolism to increased cell motility [37]. Metabolomic analyses further demonstrate that LMP1-overexpressing NPC cells exhibit significantly increased glycolytic flux, elevated pyruvate, and lactate levels [38]. This metabolic shift is attributed to the deregulation of key glycolytic genes, particularly hexokinase 2 (HK2), which promotes aerobic glycolysis, proliferation, and inhibits apoptosis [39]. High HK2 levels in NPC biopsies correlate positively with LMP1 and are associated with poorer overall survival in patients undergoing radiation therapy. Additionally, LMP-1 epigenetically reprograms the expression of several Homeobox (Hox) genes in NPC, influencing glycolysis and the TCA cycle [40]. This metabolic reprogramming confers a proliferative advantage in NPC cells and leads to the expansion of Myeloid-derived suppressor cells (MDSCs), which constitute the primary cause of tumor immunosuppression in NPC.

The effect of KSHV infection on cellular glucose metabolism has been extensively studied, with many of these investigations revealing stabilization of hypoxia-inducible factor 1 (HIF1) upon KSHV infection. Delgado *et al*. demonstrated that KSHV infection induces the Warburg effect, enhancing glycolysis and lactate production in endothelial cells by upregulating HK2 and glucose transporter 3 (GLUT3), with this metabolic shift crucial for the survival of KSHV-infected cells [41]. Subsequent study identified the KSHV-encoded G protein-coupled receptor (vGPCR) as a novel target of HIF1, contributing to increased glucose dependency and elevated lactate release in KSHV-infected cells under hypoxic conditions [42]. They Similarly, the HIF1 metabolic effector, pyruvate kinase 2 (PKM2), was found to be upregulated in KSHV infected endothelial cells, playing a crucial role in maintaining aerobic glycolysis. Furthermore, PKM2 regulates vGPCR-induced vascular endothelial growth factor (VEGF) paracrine secretion, connecting HIF1 dysregulation to both angiogenesis and tumor metabolism in Kaposi's sarcoma [43]. These findings provide insights into the synergistic effects of HIF1 and KSHV-encoded proteins, shedding light on the complex metabolic changes associated with KSHV infection.

Viral interferon regulatory factor 1 (vIRF1), a gene encoded by KSHV, emerged as a significant regulator of aerobic glycolysis. Its mechanism involves the recruitment of E3 ubiquitin ligase Kelch-like 3 (KLHL3) by vIRF1, which facilitates the degradation of heterogeneous nuclear ribonucleoprotein Q1 (hnRNP Q1) [44]. hnRNP Q1, known for its role in mRNA stabilization, was found to bind specifically to the mRNA of glycerophosphodiester phosphodiesterase domain containing 1 (GDPD1), thereby enhancing mRNA stability and subsequent protein expression. Consequently, the reduction in hnRNP Q1 levels by vIRF1 led to a decrease in GDPD1 mRNA levels, ultimately promoting aerobic glycolysis.

Conversely, disparate findings arise from studies employing different experimental models or cell types. In KSHV-transformed metanephric mesenchymal precursor cells (KMM), viral miRNAs and vFLIP downregulate GLUT1 and GLUT3, suppressing both aerobic glycolysis and oxidative phosphorylation. This adaptation enables KSHV-transformed cells to thrive in a glucose-deprived TME, supported by observations in human KS tumors [45]. These divergent results emphasize the complex and multifaceted nature of KSHV-mediated metabolic alterations, emphasizing the need for comprehensive understanding and consideration of various experimental models in elucidating the role of KSHV in cellular glucose metabolism (Fig. 1).

### Altered Mitochondrial Metabolism

The alteration of mitochondrial function is a crucial aspect of the oncogenic processes induced by gamma herpesvirus. Several mechanisms contribute to the disruption of mitochondrial homeostasis during gamma-herpesvirus-induced cancer development. EBV has been implicated in altering mitochondrial dynamics through its LMP1 and LMP2A (Fig. 1). LMP1 upregulates DNA methyltransferase 1 (DNMT1) expression, enhancing its activity and facilitating mitochondrial translocation, resulting in posphatase and tensin homolog (PTEN) silencing, activation of AKT signaling, and hypermethylation of mitochondrial DNA’s unique displacement loop. This epigenetic modification leads to the downregulation of genes associated with oxidative phosphorylation (OXPHOS) complexes [46]. Furthermore, LMP2A induces increased mitochondrial fission, accompanied by elevated levels of dynamin-related protein 1 (Drp1), a key player in mitochondrial fission. Interestingly, the enhanced mitochondrial fission mediated by LMP2A is attributed to the activation of the Notch pathway [47]. Moreover, Holmes *et al*. investigated metabolic remodeling during EBV infection of B cells, revealing increased expression of electron transport chain components and mitochondrial one-carbon metabolism. They identified EBNA2's cooperation with MYC in de novo serine synthesis as a mechanism underlying these mitochondrial alterations, collectively contributing to the development and progression of EBV-associated cancers by promoting cell migration. These mitochondrial alterations collectively contribute to the development and progression of EBV-associated cancers by increasing cell migration [48].

Mitochondrial function is recognized to play a crucial role in both latent and lytic KSHV infection. Screening with sgRNA libraries in latently KSHV infected cells unveiled genes associated with mitochondrial translation, potentially essential for the survival of latently infected cells. The knockout of genes involved in mitochondrial translation, including *MRPS34*, has been associated with decreased proliferation in KSHV-infected cells [49]. Although the mechanism remains unclear, the presence of numerous KSHV latent proteins in mitochondria suggests the need for further investigation into their role in mitochondria. Notably, KSHV microRNAs, among the known viral regulators of mitochondrial biogenesis, have been observed to stabilize HIF1 and suppress mitochondrial copy number [50]. The miRNA's expression led to decreased mitochondrial oxygen consumption and increased glycolysis. During the KSHV lytic cycle, vIRF-1 binds to the mitophagy receptor NIX, inducing mitophagy to aid in clearing mitochondria [51]. This inhibits apoptosis induced during lytic activation, ultimately facilitating productive virus replication (Fig. 1). These findings collectively emphasize the interplay between KSHV and mitochondrial dynamics in the context of both latent and lytic phases.

### Nucleotide Metabolism

**Fig. 2:**
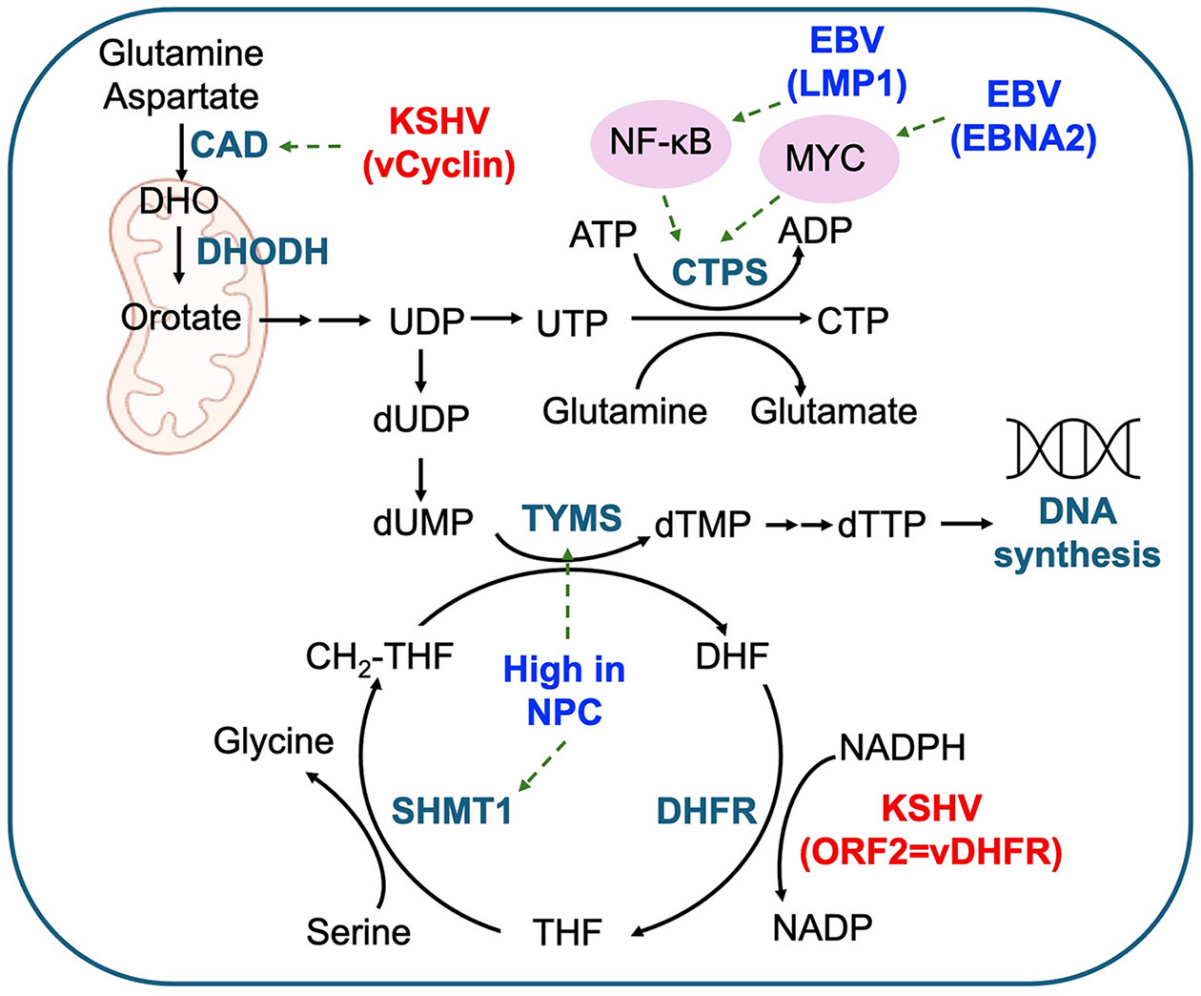
Regulation nucleotide metabolism by gamma herpesviruses. Schematic representation of EBV and KSHVmediated alterations in nucleotide metabolic pathways. EBV's LMP1 and EBNA2 upregulate CTPS enzyme expression through NF-κB and MYC. Furthermore, increased expression of TYMS and SHMT1 is observed in EBV-associated NPC. KSHV's vCyclin enhances CAD enzyme activation, subsequently activating the pyrimidine synthesis pathway, and KSHV's ORF2 functions as viral DHFR. Green dashed lines indicate activation of transcription or activity, whereas red solid lines indicate inhibition of transcription or activity.

Gamma herpesviruses intricately manipulate nucleotide metabolism pathways within infected cells to sustain viral replication and persistence, thereby contributing to dysregulated proliferation and ultimately driving tumorigenesis (Fig. 2). For instance, EBV infection induces the synthesis of cytidine nucleotide triphosphate (CTP) by upregulating the rate-limiting enzyme cytidine 5' triphosphate synthase 1 (CTPS1) [52]. This upregulation is orchestrated by viral factors such as EBNA2, MYC, and the LMP1-activated noncanonical NF-κB pathway. Targeting pyrimidine metabolism, specifically by inhibiting dihydroorotate dehydrogenase (DHODH), an enzyme supporting de novo thymidylate and CTP biosynthesis, has shown promise in restraining EBV-transformed B cell proliferation and lytic replication, suggesting therapeutic potential in EBV-associated cancers. Moreover, NPC cells demonstrate increased expression of thymidylate synthase (TYMS) and dihydrofolate reductase (DHFR), pivotal enzymes in deoxythymidine monophosphate (dTMP) synthesis, which serves as a methyl donor in nucleotide synthesis [53]. However, further investigation is needed to determine if EBV directly regulates the expression of these enzymes.

In a comprehensive study utilizing B-cell immortalization by EBV to conduct transcriptomics, Assay for Transposase-Accessible Chromatin with high-throughput sequencing (ATAC-seq), and metabolomics analyses, it was observed that EBNA1 enhances expression by binding to the enhancer regions of adenosine deaminase (ADA) and adenylate kinase 4 (AK4), resulting in increased adenosine and purine metabolism during immortalization by EBV [54]. These findings underscore the intricate modulation of nucleotide metabolism pathways by EBV infection, including the upregulation of ADA and CTPS1/2, and the dysregulation of dTMP synthesis enzymes in NPC.

In investigations into KSHV-infected cell proliferation under glutamine-depleted conditions, researchers observed heightened expression of enzymes associated with glutamine metabolism, including GLS2, GLUD1, and GOT2 [55]. Knockdown of these enzymes in KSHV-transfected cells resulted in impaired growth, underscoring the indispensability of the glutamine metabolic pathway for cellular proliferation. Intriguingly, supplementation of nucleosides restored growth, indicating the pathway's role in nucleoside biosynthesis during KSHV-induced tumorigenesis, suggesting a remodeling of this pathway for nucleotide synthesis.

Furthermore, KSHV expresses genes directly implicated in nucleotide metabolism, such as vDHFR, ORF2, which shares homology with DHFR [56] (Fig. 2). Notably, KSHV DHFR, harboring an additional 23 amino acids at the c-terminal compared to the human DHFR homolog, exhibits heightened km value and expression level. DHFR’s expression kinetics, observed within 6 to 24 h post-reactivation, hint at nucleotide metabolism involvement during the early lytic cycle. Recent studies have unveiled that vCyclin enhances dihydroorotase (CAD) enzyme activity by phosphorylating a novel serine position, underscoring its role in pyrimidine synthesis [57]. CAD orchestrates key steps in de novo pyrimidine biosynthesis, including the conversion of carbamoyl phosphate and aspartate into carbamoyl aspartate, a precursor for pyrimidine nucleotide synthesis. Consequently, treatment with CAD inhibitor reduced the pathogenesis of KSHV-induced tumors and PEL. These findings underscore the multifaceted role of KSHV in remodeling nucleotide metabolism pathways to sustain viral replication and drive tumorigenesis, shedding light on potential therapeutic targets for KSHV-associated cancers.

### Altered Amino Acid Metabolism

**Fig. 3:**
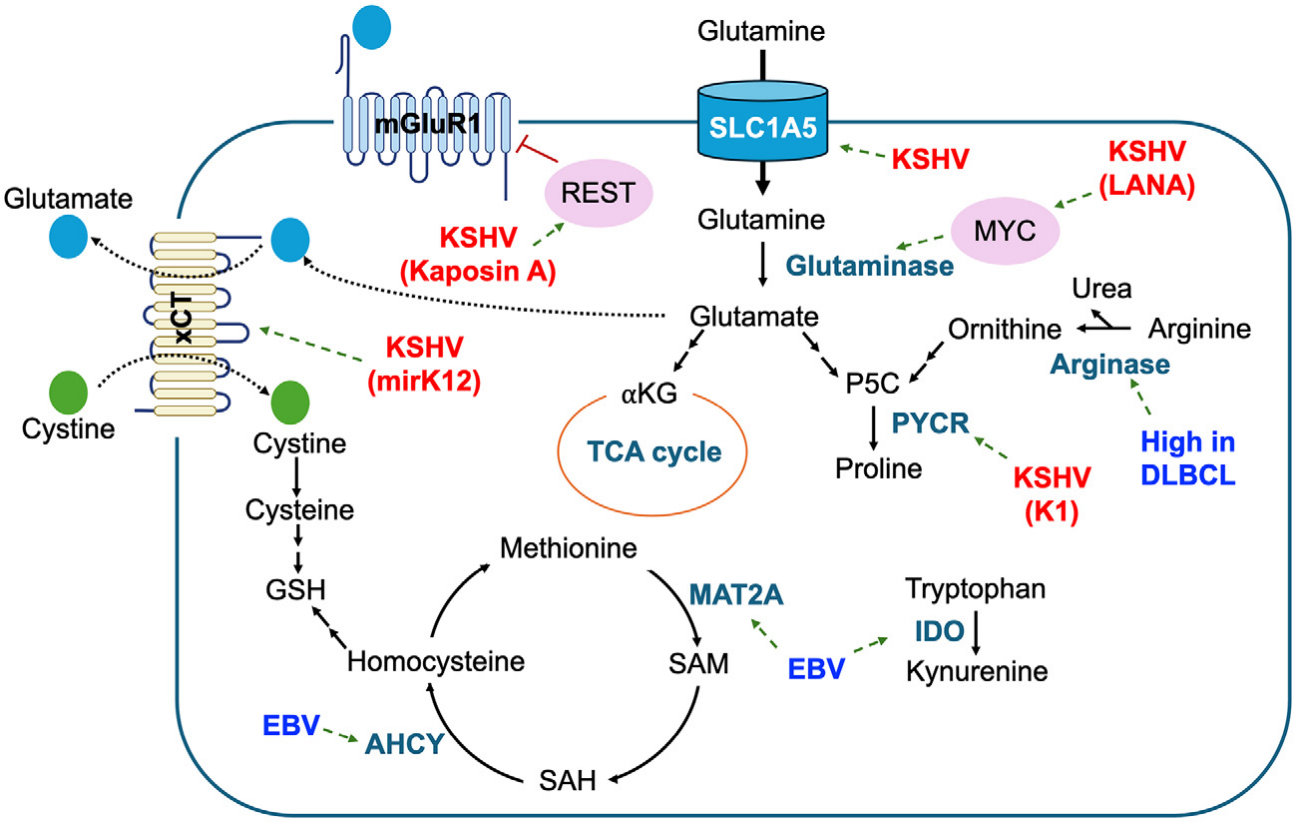
Alteration of amino acid metabolism by gamma herpesviruses. This figure illustrates key disruptions in amino acid metabolism in EBV- and KSHV-associated cancers. KSHV upregulates the glutamine transporter SLC1A5 and glutaminase, driving glutaminolysis to fuel the TCA cycle, which is vital for cell survival. Additionally, KSHV-encoded microRNA enhances the expression of the cystine/glutamate antiporter xCT, facilitating cystine import for glutathione synthesis and protecting cells from oxidative stress. EBV impacts methionine metabolism by modulating MAT2A and AHCY, influencing viral DNA and histone methylation, and altering viral gene expression. Moreover, EBV-induced overexpression of arginase-1 and IDO depletes arginine and tryptophan, impairing T cell function and promoting immune evasion.

EBV-associated cancer cells exhibit significant disruptions in amino acid metabolism, particularly affecting arginine and tryptophan pathways (Fig. 3). These alterations are pivotal for tumor cells to evade immune surveillance, promote tumor progression. Notably, in non-Hodgkin B-cell lymphoma, such as DLBCL, there is significant overexpression of arginase-1 [58]. Considering the essential role of arginine in T cell function and proliferation [59], heightened arginase levels in DLBCL may deplete arginine in the TME, impairing T cell function and creating a conducive environment for viral replication and immune evasion. Furthermore, EBV-induced expression of indoleamine 2,3-dioxygenase (IDO) in monocyte-derived macrophages (MDMs) leads to tryptophan degradation into kynurenine, impairing CD8^+^ T cell immune responses [60]. Clinical studies have reported overexpression of IDO in DLBCL, which correlates with poor treatment outcomes following rituximab and prednisone (R-CHOP) therapy [61-63].

In addition to arginine and tryptophan metabolism, EBV also alters methionine metabolism by modulating the expression of enzymes such as methionine adenosyltransferase 2A (MAT2A) and adenosylhomocysteinase (AHCY) (Fig. 3). These alterations impact viral episome DNA and histone methylation patterns, while methionine depletion affects viral gene expression patterns and the viral life cycle [13]. Notably, feeding a methionine restriction diet inhibited latency I maintenance of Burkitt tumors in mice, suggesting the therapeutic potential of exploiting the sensitivity of EBV-associated tumors to methionine restriction.

KSHV infection profoundly influences the metabolism of glutamine and glutamate (Fig. 3). Studies have revealed that KSHV-infected cells demonstrate elevated expression of glutamine and glutamate transporters and enzymes involved in glutamine metabolism, including glutaminase. Latent KSHV infection has been found to enhance glutamine uptake by upregulating the glutamine transporter SLC1A5 [64]. Subsequently, the uptaken glutamine undergoes glutaminolysis mediated by glutaminase, leading to the generation of α-ketoglutarate (αKG), which fuels the TCA cycle. Notably, experimental depletion of glutamine resulted in apoptotic cell death in latently infected cells, highlighting the dependency of KSHV-infected cells on glutamine.

Furthermore, KSHV infection induces the upregulation of glutaminase via LANA-mediated stabilization of MYC [65]. This study has suggested that KSHV Kaposin A binds to RE1-silencing transcription factor (REST), repressing the transcription of metabotropic glutamate receptor 1 (mGluR1), and sequestering it in the cytosol (Fig. 3). This action increases the expression of mGluR1, leading to enhanced uptake of glutamate. These findings underscore the intricate modulation of glutamine metabolism by KSHV infection, revealing its essential role in KSHV associated malignancies.

KSHV infection also modulates cystine metabolism, a crucial component of antioxidant defense mechanisms. In macrophages, a KSHV-encoded microRNA (mirK12) has been identified to upregulate the expression of the cystine/glutamate antiporter xCT [66]. This upregulation of xCT expression has been observed in several PEL cell lines, implicating its role in KSHV-induced tumorigenesis. Moreover, treatment of PELs with monosodium glutamate (MSG) and sulfasalazine (SASP), specific inhibitors of xCT, resulted in increased cell death accompanied by elevated levels of reactive oxygen species (ROS) [67]. These observations suggest that KSHV employs a mechanism to shield cells from oxidative stress by facilitating cystine import for the synthesis of the antioxidant glutathione.

Recently, a study utilizing 3D culture models revealed that KSHV K1 directly interacts with pyrroline-5-carboxylate (P5C) reductase (PYCR), augmenting its enzymatic activity and consequently elevating proline synthesis [68]. Within the 3D culture environment, which closely mimics the conditions of tumorigenesis with restricted nutrient and oxygen availability, enhanced proline levels induced by K1 confer protection against oxidative stress while fostering tumorigenesis through facilitation of collagen formation within the extracellular matrix (ECM). These findings underscore the importance of exploring the unique metabolic alterations induced by gamma herpesviruses further, both in 3D culture and in vivo settings.

### Lipid Metabolism

**Fig. 4:**
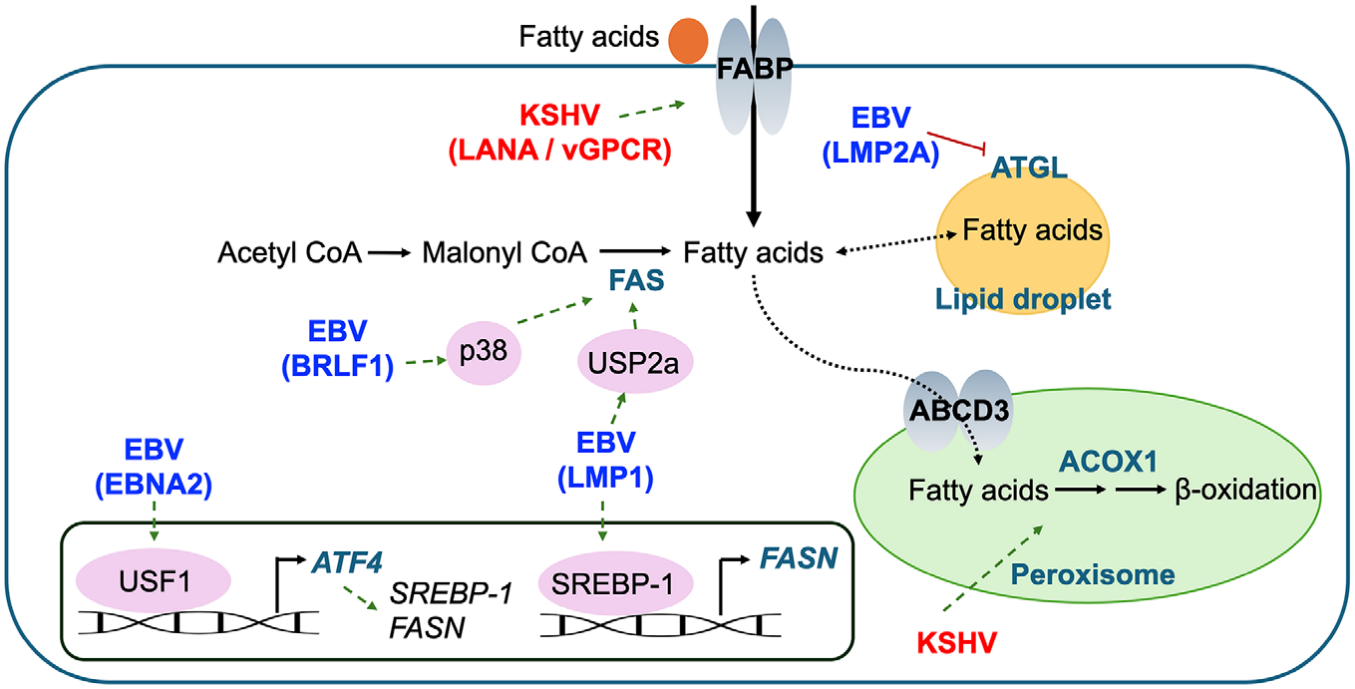
Alteration of lipid metabolism by gamma herpesviruses. This figure illustrates the crucial pathways and molecules involved in lipid metabolism in EBV and KSHV-associated cancers. For EBV, BRLF1 enhances FAS gene expression through the p38 kinase pathway, while LMP1 increases FASN expression via the SREBP-1 and USP2a, leading to increased lipid synthesis and the formation of lipid droplets. EBV further regulate lipid metabolism, supporting nasopharyngeal carcinoma (NPC) growth, with EBNA2 also inducing lipogenesis through epigenetic modifications on ATF4 promoter. LMP2A promotes lipid accumulation by inhibiting adipose triglyceride lipase (ATGL), resulting in lipid droplet accumulation. In KSHV, peroxisomal lipid metabolism is crucial for maintaining latent infection, with FABP regulation playing significant roles in the viral life cycle.

Lipid metabolism plays a crucial role in EBV-associated cancers (Fig. 4). Overexpression of EBV's BRLF1 in human keratinocytes has been shown to increase the expression of the FAS gene (*FASN*), which encodes fatty acid synthase, via the p38 kinase pathway (Fig. 4) [69]. This overexpression inhibited the expression of EBV's lytic genes when treated with a FAS inhibitor, indicating a potential link between fatty acid synthase and the EBV life cycle. Further studies revealed that EBV-encoded molecules, including the EBERs, LMP1, and EBNA2 regulate lipid metabolism through FAS to support NPC growth. Specifically, the expression of EBERs in an EBV-negative NPC cell line led to an upregulation of lipid metabolism [70]. Additionally, the presence of low-density lipoprotein (LDL) alone increased cell proliferation, while inhibiting FAS with quercetin reduced this proliferation. Overexpression of LMP1 in nasopharyngeal epithelial cells also increased *FASN* expression, lipid synthesis, and the formation of lipid droplets via the SREBP-1 pathway (Fig. 4) [71]. In human NPC samples, *FASN* expression is correlated with LMP1 expression, and high levels of *FASN* are linked to a poorer prognosis. In addition to NPC, recent studies highlight the importance of lipid metabolism in B cell lymphoma. It has been reported that fatty acid biosynthesis is increased by EBNA2 in human B cells infected with EBV [72, 73]. The proposed mechanism involves EBNA2 inducing epigenetic changes that enhance the binding of upstream stimulating factor 1 to the activating transcription factor 4 (ATF4) promoter, thereby increasing the expression of genes involved in lipogenesis, including FAS (Fig. 4) [73]. Additionally, in EBV-negative Burkitt's lymphoma cells, the expression of LMP1 was found to increase lipogenesis by stabilizing FASN through ubiquitin-specific peptidase 2a (USP2a), thus contributing to B cell immortalization [74].

In addition to the mechanism involving increased lipid synthesis through FASN, metabolic remodeling through the regulation of lipolysis pathways is also being investigated. Metabolomic comparisons between LMP2A-positive and LMP2A-negative NPCs revealed a rewiring of lipid metabolism in LMP2A-positive NPCs [75]. Specifically, these cells exhibited lipid droplets accumulation and demonstrated enhanced migration. Another mechanism noted is that LMP2A promotes lipid accumulation by reducing the expression of adipose triglyceride lipase (ATGL). Furthermore, when EBV infects adipocytes, it induces lipolysis, releasing mediators such as free fatty acids (FFAs) and glycerol, which in turn support the growth of neighboring NPCs [76].

Several omics studies have also indicated an association between lipid metabolism and KSHV infection. Proteomics and transcriptomic analyses performed on endothelial cells after KSHV infection revealed an increase in peroxisome lipid metabolism markers [77]. In these experiments, knockdown of acyl-CoA oxidase 1 (ACOX1), the main enzyme involved in the first step of peroxisomal β-oxidation, and ATP-binding cassette sub-family D member 3 (ABCD3), responsible for peroxisomal import of long-chain fatty acids, significantly increased the death of cells latently infected with KSHV (Fig. 4). This suggests that peroxisome-mediated lipid metabolism is crucial for maintaining KSHV latent infection. Furthermore, RNA-sequencing results from actual KS patient samples demonstrated alterations in genes related to lipid metabolism, indicating a connection to lipid metabolism in both experimental and clinical conditions [78].

In a more detailed mechanism, treatment with a fatty acid synthesis inhibitor induces apoptosis in KSHV-infected endothelial cells, and this cell death can be rescued by palmitic acid, suggesting that certain lipid metabolites are essential for the survival of KSHV-infected cells [79]. In KSHV-infected B cells, genes related to fatty acid metabolism were also found to be altered under hypoxic conditions. Specifically, vGPCRs and LANA regulate the fatty acid binding protein (FABP) gene family (Fig. 4) [80]. Moreover, knockdown of FABP reduced hypoxia-induced KSHV reactivation. These findings suggest that lipid metabolism plays a significant role in the KSHV life cycle, particularly under hypoxic conditions.

## Discussion

The understanding of metabolic pathway rewiring by oncogenic gamma herpesviruses, such as EBV and KSHV, offers profound insights into the mechanisms of virus-induced tumorigenesis. The intricate reprogramming of host metabolism by these viruses not only enhances their ability to persist and proliferate but also creates potential vulnerabilities that can be therapeutically targeted. By studying virus-induced metabolic remodeling, we open up new avenues for developing novel cancer therapeutics aimed at virus-associated malignancies.

The discovery of the natural compound Grifolin as a DNMT1 inhibitor, demonstrated to mitigate glycolytic flux and restore mitochondrial OXPHOS function in NPC cells, is particularly noteworthy. By counteracting the DNMT1-mediated metabolic reprogramming, Grifolin offers a potential therapeutic avenue for treating high-CpG island methylator phenotype (CIMP) tumors associated with EBV [46]. Its ability to reverse the metabolic changes induced by viral oncogenes underscores the potential for natural compounds in treating virus-associated cancers. Another promising area is the potential use of polyamine synthesis inhibitors, such as DFMO (difluoromethylornithine), which may inhibit cancer progression in KSHV-associated malignancies [81]. By targeting the polyamine synthesis pathway, which is critical for maintaining LANA expression and episome in infected cells, we can potentially inhibit the metabolic benefits that these viruses confer to their host cells.

In conclusion, the study of virus-induced metabolic remodeling presents a new perspective for developing therapeutics against gamma herpesvirus-associated cancers. By targeting the polyamine synthesis pathway, critical for maintaining LANA expression and episome stability in infected cells, polyamine synthesis inhibitors could potentially disrupt the metabolic advantages conferred by these viruses to their host cells.
